# Methods for evaluating delivery systems for scaling-up malaria control intervention

**DOI:** 10.1186/1472-6963-10-S1-S8

**Published:** 2010-07-02

**Authors:** Jayne Webster, Daniel Chandramohan, Kara Hanson

**Affiliations:** 1 Disease Control and Vector Biology Unit, London School of Hygiene and Tropical Medicine, Keppel Street, London, WC1E 7HT, UK; 2 Health Policy Unit, London School of Hygiene and Tropical Medicine, Keppel Street, London, WC1E 7HT, UK

## Abstract

**Background:**

Despite increased resources over the past few years the coverage of malaria control interventions is still inadequate to reach national and international targets and achieve the full potential of the interventions to improve population health. One of the reasons for this inadequate coverage of efficacious interventions is the limited understanding of the optimum delivery systems of the interventions in different contexts. Although there have been debates about how to deliver interventions, the methods for evaluating the effectiveness of different delivery systems have rarely been discussed. Delivery of interventions is relatively complex and a thorough evaluation would need to look holistically at multiple steps in the delivery process and at multiple factors influencing the process. A better understanding of the strength of the evidence on delivery system effectiveness is needed in order to optimise delivery of efficacious interventions.

**Methods:**

A literature review was conducted of methods used to evaluate delivery systems for insecticide treated nets, intermittent preventive treatment in pregnant women, and treatment for malaria in children.

**Results:**

The methodology of delivery system evaluations varied. There were inconsistencies between objectives and methods of the evaluations including inappropriate outcome measures and unnecessary controls. There were few examples where the delivery processes were adequately described, or measured. We propose a cross sectional observational study design with attribution of the outcomes to a specific delivery system as an appropriate method for evaluating delivery systems at scale.

**Conclusions:**

The proposed evaluation framework is adaptable to natural experiments at scale, and can be applied using data from routine surveys such as the Demographic and Health Surveys, modified by the addition of one to two questions for each intervention. This framework has the potential to enable wider application of rigorous evaluations and thereby improve the evidence base on which decisions about delivery systems for malaria control and other public health interventions are taken.

## Introduction

The efficacy of insecticide treated nets (ITNs) [1,3,4], intermittent preventive treatment in pregnant women (IPTp) [5-9] and artemisinin combination therapies (ACTs) [10-12] have been proven. However, coverage of these interventions is still low:  the most recently available data indicate that among populations at risk, only 24% of children under 5 years of age use a treated net, 20% of pregnant women receive at least two doses of IPTp, and less than 15% of febrile children receive prompt treatment with an ACT. [13]. Whether insecticide treated nets should be delivered free of charge, whether they should be delivered through the public or private sector, and whether through routine systems or campaigns is debated. The low coverage of IPTp delivered through routine antenatal care has prompted questions on whether delivery of IPTp through community based systems could increase coverage. Interventions to improve access to ACT through public, private and community based delivery systems are being implemented. Despite these debates about how to scale-up the delivery of these interventions, there has been little discussion of the methods of evaluation of the effectiveness of different delivery systems, limiting understanding of the strength of the evidence base on which the merits of different systems can be considered. 

Delivery systems have two components: (1) the channels through which a product moves from the national level to the end user; (2) the strategies applied to facilitate movement of the product from step to step of the delivery channel. The delivery channels may be within the public sector such as antenatal clinics (ANC) and campaigns, the private sector such as Licensed Chemical Sellers, or composed of a mix of the two such as voucher schemes for ITNs. The strategies to facilitate movement of the product applied to these channels include pricing policies (level of subsidy), the type or brand of product, the extent and form of training of health workers, and the formulation and packaging of the drugs. There are therefore a multitude of potential delivery systems for most public health interventions and most interventions will be delivered at any one time through more than one delivery system (different channels, strategies, or both). A public health programme such as a malaria control programme will consist of multiple products delivered through a multitude of delivery systems.

Evaluation of the effectiveness of delivery systems is essential to identify optimum delivery systems to scale up interventions. However, the methodology for evaluating delivery systems has not been well defined. Evaluations in general have focussed on the effectiveness of the intervention, or on the health impact of public health programmes. Approaches proposed for programme evaluation provide a useful framework for development of delivery system evaluations. Three types of programme evaluation have been defined based upon the strength of inference of the causal relationship between the interventions that are implemented and the outcomes. In increasing order of complexity and strength of inference, these are adequacy, plausibility, and probability evaluations [14]. The UK Medical Research Council has developed a similar framework for evaluating ‘complex interventions’ where they acknowledge the need to examine the causal pathway of interventions, which they call a process evaluation [15]. Although primarily developed from experience within high income countries, this approach may be adapted to the needs of programme evaluation within the developing country context. Examples of this approach to date have mainly been conducted within the context of Randomised Controlled Trials (RCTs). 

Although there have been calls for scaling up the delivery of effective interventions over the last few years [16,17] there have been few advances in how to assess the effectiveness of the systems required to achieve this objective. In order to optimise delivery of efficacious interventions it is critical to understand the way in which these delivery systems have been evaluated so as to assess the strength of the evidence base. Our objective is therefore to review the methods used in evaluations of delivery systems for ITNs, IPTp, and effective case management for febrile children; and, drawing on the findings from this review and upon elements of programme evaluation methodology, to develop a relatively simple approach to delivery system evaluation applicable to use by a wide range of programmes. 

## Methods

We reviewed evaluations of the delivery of ITNs, IPTp, and case management of malaria in febrile children that were found in the PubMed electronic online database (US National Library of Medicine, Bethseda, USA). Key search terms used were insecticide treated nets, ITNs, bednet, bed net, intermittent preventive treatment, IPT, IPTp, malaria treatment, malaria case management, delivery, distribution, coverage, adherence, and evaluation. The titles and abstracts were checked for relevance to the evaluation review. The reference list of each identified paper was searched for further relevant publications.

Studies were included if they involved evaluation of the delivery of ITN, IPTp or ACT through one specific delivery system, through multiple systems, or through a new delivery system. Because IPTp is almost exclusively delivered through ANC, studies of coverage of IPTp were included; in contrast, ITNs and effective case management for malaria may be delivered through a myriad of systems and therefore studies of coverage of ITNs and effective case management for malaria were excluded unless they referred to a specific delivery system(s), or a component of a specific delivery system. This review focused on the delivery channel. Thus, evaluations of delivery strategies to improve uptake and use such as pricing policies, pre-packaging of drugs, education of providers and other such strategies were excluded. For each study, the defined objective, evaluation method, primary outcome, type of control and scale were extracted.

Objectives and approaches to evaluation from the public health programme literature and the complex evaluation literature were used to develop a framework for delivery system evaluation and to discuss the limitations of the reported delivery system evaluations.

## Results

### Review of delivery system evaluations

An initial screening of 1,039 study titles identified 65 papers on ITNs, 16 on IPTp, and 54 on effective case management of malaria, that were relevant to delivery system evaluation. Upon reviewing the abstracts of these publications, 27 of the ITN, 6 of the IPTp, and 17 of the effective case management papers met the inclusion criteria. The majority of the ITN paper exclusions were due to a lack of focus on a delivery system. Excluded IPTp papers included those where health outcomes rather than coverage outcomes were reported, where the focus was on effect modifiers, for example the influence of timing of ANC visits on IPTp coverage [18], and where there was no empirical data. The reasons for exclusion of papers focused on case management were relatively wide ranging including: health rather than coverage outcomes, a specific focus on diagnosis, qualitative studies, descriptive analyses of routine data, focus on training of health workers and other effect modifying strategies.

Studies remaining in the review were divided into evaluations of new delivery systems and evaluations of existing delivery systems (including components of systems). Studies of ITN delivery included 20 evaluations of new systems, and 7 evaluations of existing systems (Additional file 1). The IPTp studies included 3 evaluations of new systems and 3 evaluations of coverage achieved through existing (ANC) systems. For effective case management 4 evaluations of new delivery systems and 13 evaluations of one or more components of existing delivery systems were identified.

#### Insecticide Treated Nets

New systems for delivery of ITNs in the public sector included routine delivery through ANC/EPI, campaign delivery integrated with other interventions (immunisations and ivermectin), and voucher systems. In the private sector, delivery has involved social marketing. Three of the studies of new systems were comparisons of two different systems, employer versus community based systems [19], sales through commercial shopkeepers versus groups of community leaders [20], and social marketing alone and together with free delivery through ANC [21]. Each of these 3 studies had a primary outcome of ‘the proportion of households with at least one net/ITN’, one was a cluster randomised controlled trial and the others used observational cross sectional surveys with comparison between geographic areas where each of the interventions were implemented.

Amongst the 20 studies of new delivery systems 16 used observational cross sectional surveys, 5 including both pre-and post delivery surveys through the new system and 11 post- only. Two of the pre- and post delivery studies used an internal control, attribution of nets in households to the system through which they were delivered [22,23]; whilst the others used external geographic controls [24], and no controls [25]. Of the post- delivery only surveys, 1 used the colour of the net to attribute it to a specific delivery system, 5 used an historical internal control, 3 used an external geographic control, and 1 used no control. Historical internal controls used questions in post ITN-vaccination campaigns on ownership and/or use of ITNs pre campaign.

One out of the 7 studies with a focus on existing ITN delivery systems aimed to evaluate two specific systems [26], two evaluated one specific system [27,28], and the remainder the mix of existing systems. Six of the studies used observational cross sectional surveys and 6 collected data in such way that it was possible to attribute nets in households to the system through which they were delivered.

#### Intermittent Preventive Treatment in Pregnancy

All 3 studies identified that evaluated new delivery systems for IPTp involved community based approaches, one integrated with ivermectin delivery [29], and 2 stand alone [30,31] (Additional file 1). All three were non-randomised intervention studies and involved external geographic controls. The three studies that evaluated delivery of IPTp through ANC were observational cross sectional studies that did not include a control. Primary outcome measures were the proportion of pregnant women who received 1, 2 or >2 doses of IPTp.

#### Case management

Four studies were identified that evaluated new delivery systems for case management of malaria. These included home management/community based delivery mechanisms [32-34] and distribution by school teachers [34]. The primary outcomes were diverse, encompassing receiving an ACT, receiving treatment according to protocol, and treatment incidence density per person-year. A similar diversity was seen in the methods and controls used in these 4 studies. One of the studies used an RCT and the other 3 used observational cross sectional surveys post-delivery only. Amongst the cross sectional survey evaluations 2 used external geographic controls and one did not use a control. The 13 evaluations of existing delivery systems for malaria case management were diverse in their objectives, and primary outcomes ranged from evaluations of quality of case management after a policy change to studies on adherence. However, the majority of the studies used observational cross sectional surveys either at health facilities or the household, and did not use controls.

### A framework for delivery system evaluation

Our review found that evaluations of delivery systems for malaria control interventions have been diverse in their objectives, outcomes measured, methods and controls used. The type of control used is a major factor in determining the strength of inference that the outcomes were due to the delivery system. However, different types of controls introduce different levels of complexity and resource needs (research costs).

 We identified only 3 published evaluations of delivery systems for malaria control interventions that had taken place at the national level [22,35,36]. An effective delivery system (or mix of delivery systems) should be able to deliver the intervention to the entire target group, on a large scale. There should be no disparities in the coverage of the intervention between sub groups of the target population, for example to different socio-economic groups. The effectiveness of the delivery system in reaching different population groups is likely to vary, as is its relative effectiveness at the small and large scale. We explore the evaluation elements required and propose a framework for the evaluation of delivery systems for malaria control interventions.

### Objectives and outcome measures of delivery system evaluations

**Figure 1:**
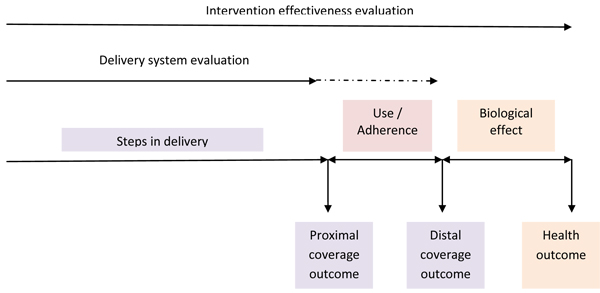


The first step in designing a delivery system evaluation is to define the purpose and objectives of the evaluation (Table 1). Generally the effectiveness of a delivery system in reaching the maximum target population is assessed. Thus the primary outcome measure in a delivery system evaluation is coverage of the intervention achieved through the specific delivery system(s) (Figure 1). Secondary outcomes include tempo (how quickly a system can reach high coverage), equity (the socio-economic distribution of coverage achieved), cost and/or cost-effectiveness, and others. Primary outcome measures are classified further into proximal and distal outcomes (Table 2). Proximal outcomes are those intrinsically linked to delivery such as ownership of an ITN, delivery of a dose of IPTp, and delivery of an ACT and therefore measure the effectiveness of the causal chain or the intermediate steps within the delivery system. Distal outcomes relate to use of the intervention once it has been delivered, such as use of an ITN by the target group, and adherence to an ACT regimen, all of which may be mediated by factors other than the delivery channel (e.g. the delivery strategies but also other factors). If IPT is delivered by directly observed therapy (DOT) within the ANC then this is a proximal outcome. However, if IPTp is prescribed rather than given by DOT (or given to be taken later) then use of the IPTp is a distal outcome (Table 1). Thus the distal outcomes evaluate the steps in the causal chain that are not entirely within the delivery system.  Measuring health outcomes (impact) is not essential unless there is a plausible reason that identical coverage of the intervention achieved via different delivery systems would result in different health impacts.

**Table 1:** Steps in designing a delivery system evaluation

	Examples	Comments
**Determine the purpose of the evaluation**	- To evaluate a new delivery system for ITNs - To evaluate a new delivery system for IPTc - To assess the delivery of IPTp through ANC	Evaluation of a new delivery system for an existing intervention requires a pre-post survey with attribution of nets by source. A process analysis is required to assess the outputs at each intermediate step in the causal chain of delivery

**Select the evaluation method**	- cross sectional pre-post survey with attribution of outcomes by source of intervention - cross sectional post intervention survey with attribution of outcomes by source of intervention - cross sectional post only survey with no control	For evaluation of a new delivery system for an existing intervention a pre-post survey with attribution of outcomes by source would provide causality for proximal indicators and plausibility inference for distal indicators.

**Define the outcome indicators**	- the proportion of children under 5years using an ITN - the proportion of children under 5 years taking a full course of IPT - the proportion of pregnant women who attend ANC receiving at least 2 doses of IPT	The primary outcome indicator may be a distal or proximal indicator

**Define the pathway of delivery**	- include several proximal and more than one distal steps - includes several proximal and one distal steps - the evaluation terminates at proximal steps	The number of steps varies with interventions and with delivery systems.Many pathways are linearNot all pathways are linear

**Characterise the contextual factors **	- malaria transmission levels - structure of the health system - socio-demographics of the population	Disaggregate outcomes by contextual factorsDescribe contextual factors

**Table 2:** examples of proximal and distal coverage outcomes for three malaria control interventions

Intervention	Delivery details	1^st^ level of proximal coverage outcome	Subsequent proximal coverage outcomes	Distal coverage outcome
ITNs	Direct delivery through ANC	Proportion of households owning at least one ITN delivered through ANC		Proportion of the target group who slept under an ITN delivered through ANC

IPTp	Directly Observed Treatment (DOT)	Proportion of pregnant women taking 2 doses of IPTp		None
	Dose given but not DOT	Proportion of pregnant women given 2 doses of IPTp		Proportion of pregnant women who take 2 doses of IPTp
	SP prescription given	Proportion of pregnant women given 2 IPTp prescriptions	Proportion of women who collect the SP	Proportion of pregnant women who take 2 doses of IPTp

ACTs	Delivery to febrile children through health facilities	Proportion of febrile children accessing public sector health facilities for whom ACT is prescribed	1. Proportion of carers of children prescribed ACTs who collect the ACT (correct number of tablets)2. Proportion of carers of children who are explained the dosing regimen	Proportion of children given ACTs who take the correct dosing regimen (number of tablets each time, number of times each day, number of days)

The second step in conducting a delivery system evaluation is to clearly characterise the pathway of the delivery system and to define the proximal and distal coverage outcomes. For example, IPTp may be intended to be delivered as DOT, however, if there is no water in the health facility the woman may be given the SP to take at home; similarly, stock outs of SP may result in the woman being given a prescription for the SP. The absence of SP in the ANC and the absence of water in the ANC are independent ‘implementation related factors’ [37]. The probability of a pregnant woman receiving 2 doses of SP-IPT will therefore be the product of these events in the pathway of the IPT delivery system.

It is plausible that the relationship between the proximal coverage outcome and the distal coverage outcome would depend upon the system through which the intervention is delivered. For example, pregnant women and children under 5 years receiving free ITNs delivered through ANC may be more, or less, likely to use the ITNs than those mothers and children obtaining ITNs delivered through social marketing in the retail sector, or from the informal private sector. Children may be more or less likely to be given a full dose of ACT (correct number of tablets each time, correct number of times per day, correct number of days) if their carers get the drug from the public sector than from the private retail sector.

The relationship between distal outcomes and health impact is dependent upon the intervention itself and upon the context. Similar distal coverage outcomes of an intervention could result in different health impacts among different population groups including different age groups, those living in different transmission intensity areas, and different socio-economic groups. However, it is unlikely that this difference in health impact is due to the system through which the intervention was delivered. For example, if the population of one district all use an ITN (distal coverage outcome) on the same nights for the same number of hours during a one year period the health impact may differ between children 0 to 2 years of age, children 3-5 years of age, older children, and adults, but this difference in health impact would not be related to the system through which the ITN was delivered. In terms of the causal chain of the intervention, the relationship between health impacts at a given level of use is not directly linked to the delivery system, whose impact is exerted upon proximal outcomes.

In summary, delivery system evaluations should 1) determine the purpose of the evaluation, 2) select the evaluation method, 3) define the outcome indicators (proximal, distal or both) 4) define the pathway of delivery, and 5) characterise the contextual factors. Each of these steps in the evaluations may be undertaken for unique delivery systems (where only one system is operating) and for specific multiple delivery systems within a mixed system.

### Attribution of coverage outcomes to a specific delivery system as an internal control

If an intervention is delivered through a unique system, then coverage outcomes can be directly attributed to this specific delivery system. For example, Intermittent Preventive Treatment for Infants (IPTi) is only delivered through the Expanded Programme on Immunisation (EPI). Where an intervention is delivered through more than one system, then further methods are needed to attribute the coverage achieved by each system. This has been done for mosquito nets based on whether the net was treated or not [38], upon the source or delivery point of the net [39] and whether a voucher was used in the purchase of the net [22,23]. A single cross sectional survey may be used to assess the relative proportion of coverage of an intervention that is due to one specific delivery system, or to all known delivery systems. A new delivery system introduced within existing multiple systems, can be evaluated by attributing the proportion of coverage to each delivery system pre and post implementation of the new delivery system.

Attribution to specific delivery systems requires a simple way of matching the coverage to the system through which it was achieved. All malaria control interventions have a point at which they are delivered to the users. The coverage of an intervention can be matched to a specific delivery system by identifying the delivery point at which the recipient received the intervention. This can be done by adding a few questions to cross sectional surveys. For example “where did you get this net” or “where did you get these medicines for your child”? This method assumes that the alternative delivery systems in operation for an intervention do not share the delivery point of the system that is being evaluated. However, if there are instances where two delivery systems share a delivery point (for example a voucher system for ITNs, and subsidised delivery of ITNs through ANC clinics) then further questions will be needed to distinguish the two.

### Assessing proximal coverage outcomes

Evaluations should consider the simplest way of achieving their objectives whilst maintaining internal validity of the methods used, and the external validity of the findings.

#### Internal validity

An internally valid evaluation minimises random and systematic errors due to chance, bias, and confounding [40]. Data collection methods for delivery system evaluation should be internally valid and should apply statistical methods in the analysis to assess random errors and adjust for any potential confounding effects. Well designed RCTs have strong internal validity as they minimise both random and systematic errors. However, assessment of a number of delivery systems using an RCT would be prohibitively complex and expensive, and potentially infeasible. Cross sectional observational studies are generally of weaker internal validity than are RCTs. However, using structured random sampling techniques to select an adequate number of appropriate units can reduce selection bias and random errors, and data on potential, confounding factors can be collected and accounted for in the analysis.

#### Inference

Where an intervention is delivered through a single system, then the proximal coverage outcomes can be directly attributed to this delivery system and it is appropriate to infer that the delivery system had a causal relationship to the proximal coverage outcome. However, unless the intervention is new, then it cannot be assumed that it is delivered through only one system. In this situation either formative work must be undertaken to ensure that there is only one delivery system in operation, or a question on source should be included in the evaluation. Where a new delivery system is evaluated within the context of multiple existing delivery systems, if the relative proportion of the proximal coverage outcome is attributed to each of the delivery systems, then a plausibility statement can be made on what proportion of the outcome was due to the new delivery system. In this type of evaluation, the existing delivery systems are acting as internal controls and thus it is possible to infer that the changes in coverage were due to the new delivery system, above and beyond the influence of other external factors. As proximal outcomes, such as coverage of IPTp in any delivery system using DOT, are direct outcomes of the delivery systems, the contextual factors that play a role in this outcome are integral to all delivery systems that are assessed. These contextual factors should be described and their effect on the coverage outcome should be assessed where possible.

#### External validity

The findings of a controlled trial may have limited external validity even with respect to the population in the area in which the trial was conducted. Well conducted cross sectional observational studies will have good generalisability to the population from which they were sampled. Therefore if a survey is undertaken at the national level, then the findings are generalisable at the national level. Characterisation of the contextual factors that are present in the area of implementation will help to inform a judgement as to the other geographic areas to which the findings may be generalised.

### Assessing distal coverage outcomes

Distal coverage outcomes measure the use of an intervention by the target population, and they are the primary link between intervention coverage and health impact.

#### Internal validity

Distal coverage outcomes are measured in the same way as proximal coverage outcomes through RCTs or cross sectional observational studies. The methodological issues in the internal validity of proximal coverage outcomes mentioned above would therefore apply to that of distal coverage outcomes.

#### Inference

The effect of the “user related factors” that influence the distal coverage outcomes may vary depending on the way the intervention was delivered (implementation related factors), or there may be external factors that modify the distal coverage outcomes. The effect of implementation related factors on the distal coverage outcome may be assessed by measuring the relative dose-response relationship (although care must be taken to assess any selection biases in the dose received) [14,40]. For example the relationship between ownership and use of ITNs from specific delivery systems can be measured. External factors are more difficult to define and to assess. For example, the proportion of those owning an ITN who use it may depend upon factors such as season (temperature), levels of biting nuisance, housing characteristics, irrespective of the system through which they got the ITN. If use of ITNs amongst those owning them is attributed to specific delivery systems then the other delivery systems act as an internal control for external factors. This would enable a plausibility inference as to the observed association between ITN use and a specific delivery system.

#### External validity

There are factors additional to those confounding proximal coverage outcomes that may confound the relationship between the delivery system and the distal coverage outcomes. As in the case of proximal coverage outcomes, the findings of a controlled trial for distal coverage outcomes may have very limited external validity. Again the external validity of cross sectional observational studies depends upon a population level representative sampling scheme and upon characterisation of the implementation context.

### Assessing steps in the causal pathway of delivery

In an effective delivery system, the intervention will progress through each intermediate step with minimal loss, for example, all febrile children prescribed an ACT will receive the correct number of tablets. It is likely in practice however, that there will be some loss at each stage of the delivery process. For example, some febrile children prescribed an ACT will be given artesunate monotherapy, or insufficient tablets to complete effective treatment. In order to assess the steps on the causal pathway of delivery of an intervention it is necessary to define these steps. The evaluation can then be designed to assess the proportion of the population that progress successfully through each step. Often during implementation, variations to the causal pathway will be introduced. These may involve health worker strategies for coping with drug stock-outs such as writing a prescription and sending the child to another health facility or the private market. Where important blockages in the steps of the causal pathway are identified, for example those eligible for an intervention not being offered it [23], then further research is needed to identify the reasons why the problems occur. Once the problems have been identified steps may be taken to prevent them reoccurring. Factors that impact upon the delivery system are termed implementation related factors, and they function as effect modifiers.

### Assessing the factors that influence the relationship between proximal and distal coverage outcomes

Factors influencing the relationship between proximal and distal outcomes can be related to 1) delivery system, 2) the intervention, 3) the target group, and 4) context and factors external to the delivery system. For example the delivery point of an ITN, and the accompanying information and education, is likely to influence household ownership, but may also affect use of ITNs that are already owned.

For example, the delivery point for an ITN is more likely to influence household ownership than use of ITNs. It is possible however, that the strategies that make up the delivery system may influence use, for example, whether the ITN was given free of charge or was purchased. Information exchanged during delivery may also affect use or patterns of intra-household use. The nature of the ITN, such as its shape, material or colour may influence whether it is used. Target group characteristics include number of household members, number of ITNs owned, education of the household head and their spouse, and socio-economic status. External factors include season, levels of biting nuisance, cultural norms etc. The external factors will have an equal influence on households with ITNs delivered through different systems and therefore do not necessarily need to be measured, but should be described.

### Other factors influencing selection of method

#### Policy status

Depending upon the policy status of the intervention to be delivered, it may not be possible to include control groups to whom the intervention will not be delivered. Where an intervention is part of the national policy it is unethical and likely to be politically impossible to systematically exclude sub groups of the population from a particular delivery system. In this situation, delivery system evaluations would therefore need to compare outcomes among those receiving the intervention through one delivery system compared to an alternative system, or through a combination of the two.

Cross sectional observational studies are not limited by whether an intervention is policy or not. As they are able to use internal controls, cross sectional observational studies are applicable to evaluating the role of alternative delivery systems in operational contexts, and to evaluating proximal and distal outcomes of interventions. For example, evaluation of the delivery of ITNs through ANC in an area with ongoing delivery of ITNs through social marketing would assess the relative proportion of the coverage due to delivery through ANC compared with that achieved through social marketing, and other systems in operation such as the formal and informal private sectors.

#### Scale

RCTs are not usually conducted at scale because they are very expensive, prohibitively difficult, and randomisation to intervention and control groups on a large scale is practically and politically difficult. The complexity and level of feasibility of evaluating at scale depends upon the type of RCT. The most feasible would be to randomise relatively large geographic areas , such as districts or sub-districts, and allocate these to different delivery systems [40].

Where a delivery system is in operation at the national level, pre and post implementation cross sectional observational studies can be undertaken using standard sampling techniques to provide coverage estimates attributable to the delivery system being evaluated that are representative at the national level.

## Discussion

In evaluating the effectiveness of a delivery system we wish to know the proportion of the target population that have been reached with the intervention, and whether there are any geographic or socio-economic disparities in coverage. Where coverage is less than required, we also need to know where on the causal pathway of intervention delivery the problems are located. In order to provide the link between delivery system effectiveness and the health outcomes we should also assess the use or adherence to the intervention by the target population. Assessment of health outcomes is not necessarily required for delivery system evaluations. If a need to measure health outcomes is identified, then an evaluation of the intervention itself is required, which may or may not, include delivery system effectiveness as a composite element of the evaluation.

Different approaches to evaluation of delivery systems for ITNs as compared with IPTp and effective case management are likely to have been influenced by the nature of the intervention. Mosquito nets to which insecticide treatment is added to produce an ITN have been household goods in most of Africa, but particularly in West Africa, for many years. They have therefore been delivered through a variety of systems, and there is no innately obvious appropriate system through which they should be delivered to reach the whole target population. Consequently, delivery system evaluations have covered a range of options, and evaluation methods have generally been aimed at assessing the relative coverage attributable to existing delivery systems, or to new ones within the context of those existing, and to the population groups targeted. IPTp and effective case management, however, are drug based, and national policy usually dictates that they should be delivered through public sector health facilities, and often in combination with other delivery systems, for example policies in many countries now allow delivery of ACTs through the private sector. With a target group of pregnant women, ANC is the obvious delivery system for IPTp. Alternative delivery systems would be required if the target population were not being reached through ANC.

There is unlikely to be a situation where ITNs are delivered by one system alone. As such, there will always be the need to attribute outcomes to specific delivery systems and the possibility of using the other delivery systems as internal controls. This should negate the need for external (geographic) controls and randomization. For example, in the cluster randomized trial of introduction of a new delivery system for ITNs within the context of an existing delivery system by Mueller *et al *[21], the use of a randomized control introduced unnecessary complexity. Rather than using a cross sectional survey pre and post RCT to assess the proximal coverage outcome, cross-sectional surveys pre and post implementation in routine operational conditions with attribution of the proximal coverage outcome to the specific ITN delivery systems would have been sufficient to achieve the objective of this evaluation. This approach would provide useful information about the effectiveness of each of these systems, and whether they are complementary or competing.

Other ITN delivery system evaluations have used longitudinal cohort studies and observational cross sectional surveys with attribution of the outcomes to the system through which they reached the target population. The outcomes of these studies have been both proximal and distal coverage outcomes. Although it has not always been noted within the reports, these studies have demonstrated either causality for the proximal outcomes or at the least strong plausibility that the distal outcomes were due to the delivery systems being studied.

Few studies have described in sufficient detail the structure of the delivery systems being evaluated, and only in a minority has the causal pathway been described [20,41] and several proximal outcomes assessed [22,23,42,43]. Generally, very little information is provided on the causal pathway of the delivery of the intervention. Only by describing the causal pathway is it possible to identify the implementation effect modifiers and to ensure that these are included in evaluation. There is perhaps a greater tendency towards assessment of health outcomes (that is evaluation of the effectiveness of the intervention) than to greater exploration of the delivery system and its enabling and disabling factors.

Evaluations of the delivery of IPTp have mostly involved the use of non-randomized controls. These studies have involved delivery of IPTp through 2 or more systems. As for the delivery of ITNs, where doses of IPTp are attributed to specific delivery systems, external controls are unnecessary. Non-randomized external geographic controls may be subject to a myriad of confounders which influence the relationship between the delivery system and the outcomes. Where a new delivery system is implemented it is essential that evaluations provide information on the period of time between implementation and evaluation as the tempo of different delivery systems in achieving increased coverage with interventions will differ.

There have been few evaluations of new systems for delivering effective case management to febrile children and there has been no common methodological approach. Study designs are complicated by the choice of whether to include [inclusion or exclusion of] diagnosis for the presence of malaria parasites.

In summary, where an intervention is delivered through two or more systems, attribution of outcomes to the specific delivery systems will enable a causal inference that proximal outcomes were due to the system through which they were delivered, and a plausible inference for distal outcomes. Each delivery system functions as an internal control for the other systems and as such is affected by existing external contextual factors. Implementation effect modifiers are internal to each specific delivery system. The causal pathway of the delivery system should be defined so that proximal outcome indicators at each step can be determined and assessed. Likely effect modifiers at each of these steps may then also be identified and included within the evaluation. If health outcomes need to be measured then an intervention effectiveness study should be conducted rather than a delivery system evaluation. This methodology may be applied to the conventional cross sectional surveys addressed here, or could also be applied to models of continuous surveys as recently recommended by Rowe *et al *[44].

## Conclusions

The practical implications of this evaluation framework are that observational cross sectional surveys can be implemented on a large scale, and applied easily to either the evaluation of new delivery systems, natural experiments, or to evaluation of the current situation. The addition of one to two questions per intervention to national surveys such as the demographic and health surveys would enable such evaluations at little or no extra cost.

## Competing Interests

The authors declare that they have no competing interests.

## Authors’ contributions

JW conceptualized the paper, conducted the review and wrote the first draft. DC and KH critiqued the proposed methodology and revised the draft. 

## Supplementary Material

Additional file 1 Summary of delivery system evaluations Click here for file
