# Supplementary material for: Methods for evaluating delivery systems for scaling-up malaria control intervention
Source: BMC Health Serv Res. 2010 Jul 2;10(Suppl 1):S8. doi: 10.1186/1472-6963-10-S1-S8 (PMC2895752; doi:10.1186/1472-6963-10-S1-S8)
Supplement: Additional file 1 — Summary of delivery system evaluations [file 1472-6963-10-S1-S8-S1.docx]

Additional file 1: Summary of delivery system evaluations

| **Delivery system** | **Primary outcome** | **Design** | **Scale** | **Country** | **Reference** |
| --- | --- | --- | --- | --- | --- |
| **ITNs: a) New systems** |  |  |  |  |  |
| Retail sector social marketing | Proportion of households that own at least one net.  Proportion of children under 5 who slept under a net the night before the survey | Observational cross sectional survey (post only)  Internal control: attribution^1^ by colour of net | 1 district | Malawi | Holtz *et al* 2002 [45] |
| Routine ANC | Proportion of nets procured that were distributed to pregnant women within 12 weeks | Interviews with health staff, ANC attendees and health facility audits  No control | 35 districts | Kenya | Guyatt *et al* 2002 [43] |
| Routine delivery through clinics and NGOs | Proportion of families who purchased a net | Observational cross sectional survey (pre-, mid, and post)  No control^2^ | 12 villages | Afghanistan | Rowland *et al* 2002 [25] |
| Routine ANC | Proportion of pregnant women who use the free ITN | Longitudinal  No control | 2 districts | Kenya | Guyatt et al 2003[42] |
| Mass distribution in Internally Displaced Person camps | Proportion of households with at least one ITN  Proportion of persons sleeping under a net | Observational cross sectional survey (post only)  No control | 22 camps | Uganda | Spencer *et al* 2004 [46] |
| ITN-measles vaccination campaign | Non-standard indicators  Proportion of caregivers who reported their youngest child had slept under an ITN received during the campaign | Campaign exit interviews + observational cross sectional survey (post only)  Historical internal^3^ control | 1 district | Ghana | Grabowsky *et al* 2005 [47] |
| ITN-measles vaccination campaign | Non-standard indicators (children’s ITN ownership) | Observational cross sectional survey (post only)  Historical internal control | 5 districts | Zambia | Grabowsy *et al* 2005 [48] |
| Community based social marketing and unassisted private sector | Proportion of households with at least one net | Observational cross sectional survey (pre- and post)  External geographic control | Intervention 6 villages, control 1 division | Tanzania | Kikumbih et al 2005 [49] |
| Lymphatic filariasis mass drug administration programme | Proportion of households with at least one ITN | Observational cross sectional survey (post only)  Historical internal control | 1 LGA in each of 2 states | Nigeria | Blackburn *et al* 2006 [50] |
| ITNs, Vitamin A, measles vaccination and mebendazole campaign | Proportion of households with an under 5 owning at least one bednet and/or ITN | Observational cross sectional survey (post only)  Historical internal control | 1 district | Tanzania | Skarbinski et al 2007 [51] |
| Commercial shopkeepers and community leaders | Proportion of households with at least one net | Observational cross sectional survey (post only)  External geographic control | 20 sites in 9 districts | Mozambique | Brentlinger *et al* 2007 [20] |
| Employer and community based | Proportion of households with at least one net | Observational cross sectional survey (post only)  External geographic controls | 4 ‘sites’ | Kenya | Wacira *et al* 2007 [19] |
| Public sector clinics and village based volunteers | Non-standard indicators.  Access to nets by socio-economic groups | Observational cross sectional survey (post only)  External geographic control | 3 districts | Zambia | Agha *et al* 2007 [52] |
| ITN-polio vaccination & Vitamin A campaign | Proportion of households owning an ITN | Observational cross sectional surveys (2 x post campaign)  Historical internal control | National | Niger | Thwing *et al* 2008 [35] |
| ANC and retail sector social marketing programme | Proportion of households owning at least one Serena ITN | Cluster randomised controlled trial  Intervention: ANC + social marketing  Control: social marketing only | 1 province | Burkina Faso | Muller *et al* 2008 [21] |
| ITN voucher scheme | Proportion of pregnant women using an ITN | Observational cross sectional surveys (pre and post surveys)  Internal control: attribution^1^ of nets by source | National | Tanzania | Hanson *et al* 2009 [22] |
| Routine ANC | Proportion of pregnant women using an ITN | Cohort study  Attribution of nets: based on type | 1 clinic | Democratic Republic of Congo | Pettifor *et al* 2009 [53] |
| Routine immunisation services | Proportion of children 12 to 23 months using an ITN | Observational cross sectional surveys (pre and post surveys)  External geographic control | 3 districts | Malawi | Mathanga *et al* 2009 [24] |
| ITN-measles vaccination campaigns | Proportion of households with any mosquito nets. | Observational cross sectional surveys (post only)  No control | 16 Local Government Areas (LGA) | Nigeria | Afolabi *et al* 2009 [54] |
| ITN voucher scheme and routine ANC direct delivery of ITNs | Proportion of households with at least one mosquito net | Observational cross sectional surveys (pre and post surveys)  Internal control: attribution of nets by source | 2 regions | Ghana | Webster *et al 2010 In press [23]* |
| **ITNs: b) Existing systems** |  |  |  |  |  |
| Multiple delivery strategies | Proportion of households with at least one net.  Proportion of households newly owning a net from the initiative | Observational cross sectional survey (single survey + sales records  Internal control: attribution of nets by source | 6 villages + 1 town | Tanzania | Fraser-Hurt *et al* 1998 [55] |
| Community based delivery | Proportion of children under 5 years not under an ITN in ITN owning households | Observational cross sectional survey (single survey)  No control | 3 *zobas* | Eritrea | Macintyre *et al* 2006 [28] |
| Multiple delivery strategies | Proportion of children who slept under a net the night before the survey  Proportion of nets that were from the retail sector | Observational cross sectional survey (single survey)  Internal control: attribution of nets by source | 4 districts | Kenya | Noor *et al* 2006 [27] |
| ITN-measles campaign + routine clinic delivery + commercial market | Non-standard indicators (children’s ITN ownership) | Observational cross sectional studies (single survey)  Internal control: attribution of nets by source | 1 district | Ghana | Grabowsky *et al* 2007 [26] |
| Multiple delivery strategies | Proportion of children <5 years who slept under any net the night before the survey | Longitudinal cohort study  Internal control: attribution of nets by source | 4 districts | Kenya | Noor *et al* 2007 [39] |
| Multiple delivery strategies | Proportion of nets used by infants and young children (12-59 months) | Observational cross sectional survey (single survey)  Internal control: attribution of nets by source | 31 villages | Tanzania | Khatib *et al* 2008  [56] |
| Multiple delivery strategies | Proportion of children <5 years who slept under any net the night before the survey | Observational cross sectional survey (single survey)  Internal control: attribution of nets by source | 1 district | Tanzania | Bernard *et al* 2009 [57] |
| **IPTp: a) New systems** |  |  |  |  |  |
| Multiple community based strategies | The proportion of pregnant adolescents and primigravidae who received 2 doses of IPtp | Non-randomised intervention study  Control: health facilities  Intervention: community based systems | 25 parishes | Uganda | Mbonye *et al* 2007 [31] |
| Community Directed Distributors (CDD) of ivermectin | The proportion of pregnant women who received 2 doses of IPTp | Non-randomised intervention study  External geographic control  Intervention: ANC + CDDs  Control: ANC | 2 districts | Uganda | Ndyomugyenyi *et al* 2009 [29] |
| Community based delivery | 1) The proportion of pregnant women who received >2 doses of SP-IPT  2) The proportion of pregnant women attending ANC at least twice during the current pregnancy | Non-randomised intervention study  External geographic control  Intervention: community health workers (CHWs) give information + SP  Control: CHWs give information | 14 intervention and 12 control villages | Malawi | Msyamboza *et al* 2009 [30] |
| **IPTp b) Existing systems:** |  |  |  |  |  |
| ANC | Trends in proportion of pregnant women taking 1 and 2 doses of IPTp | Observational cross sectional household and facility surveys (3 consecutive years)  No control | National | Tanzania | Marchant *et al* 2008 [36] |
| ANC | Proportion of pregnant women receiving 1 or ≥2 doses of IPTp | Observational cross sectional survey (household level)  No control | One district | Uganda | Kiwuwa *et al* 2008 [58] |
| ANC | Proportion of ANC attendees receiving at least one dose of IPTp | Observational cross sectional survey (single survey)  No control | 1 Local Government Area | Nigeria | Akinleye *et al* 2009 [59] |
| **Effective treatment: a) New systems** |  |  |  |  |  |
| Community based | Proportion of visits for which guidelines for dipstick use and treatment were followed | Observational cross sectional surveys  Historical control + external geographic control | 2 villages | Brazil | Cunha *et al* 2001 [34] |
| School teachers | Proportion of correctly diagnosed cases that were treated according to the protocol | Observational cross sectional survey (post only)  No control | 2 sub-districts | Ghana | Afenyadu et al 2005 [60] |
| Outreach clinics and Village Malaria Workers | Proportion of respondents with fever in the last 3 weeks who received an ACT by delivery system | Observational cross sectional survey (single survey)  External geographic control | 23 villages | Cambodia | Yeung et al 2008 [33] |
| Home based delivery | Treatment incidence density per person-year | Randomised controlled trial  Control: standard care  Intervention: home management | 1 parish | Uganda | Staedke *et al* 2009 [32] |
| **b) Components of systems** |  |  |  |  |  |
| Multiple delivery strategies | Type of drug dispensed by source | Observational cross sectional survey (single survey)  Internal control: attribution of treatments by source | 4 districts | Kenya | Amin et al 2003 [61] |
| Public sector | Self reported adherence to artemether-lumefantrine | Observational cross sectional survey (single survey)  No control | 7 ‘malaria sections’ | South Africa | Barnes et al 2005 [62] |
| Community Drug Distributors | Proportion of caretakers who adhered to the dose of antimalarials given | Observational cross sectional survey (single survey)  No control | IDP camps in 1 district | Uganda | Kolaczinski et al 2006 [63] |
| Public and mission health facilities | Proportion of patients undergoing malaria diagnostic procedures and receiving anti-malarial treatment | Observational cross sectional survey (single survey)  No control | 4 districts | Zambia | Hamer et al 2007 [64] |
| Multiple delivery strategies | Proportion of children with (a symptom based) uncomplicated malaria who were treated with AS+AQ | Observational cross sectional survey (single household survey)  Internal control: attribution of treatment to source | 1 province | Burundi | Gerstl et al 2007 [65] |
| Government and mission facilities | Proportion of children with uncomplicated malaria treated with (AL) | Observational cross sectional study (single health facility survey)  No control | Four districts | Zambia | Zurovac et al 2007 [66] |
| Government and private-not-for-profit facilities | AL prescribing, dispensing and counselling practices | Observational cross sectional study (single health facility survey)  No control | Four districts | Uganda | Zurovac et al 2008 [67] |
| Government facilities | Proportion of children who needed AL who were given it | Observational cross sectional study (single health facility survey)  No control | Four districts | Kenya | Zurovac et al 2008 [68] |
| Multiple delivery strategies | Proportion of children and adults receiving prompt and appropriate antimalarial treatment | Observational cross sectional survey (single survey)  Internal control: attribution of treatment to source | 2 districts + 1 town | Tanzania | Hetzel et al 2008[41] |
| Retail sector | 1) Proportion of shopkeepers who mentioned the correct child-dosage of SP  2) the odds that a mystery shopper would receive an appropriate treatment | Observational cross sectional survey (single retail outlet survey + mystery shopper survey)  No control | 2 districts | Tanzania | Hetzel et al 2008 [69] |
| Public sector health facilities | Anti-malarial treatment practices | Observational cross sectional survey (single survey)  No control | 4 districts | Kenya | Zurovac et al 2008 [70] |
| Public and private health facilities | Quality of malaria case management | Observational cross sectional survey (single survey)  No control | 1 Province | Angola | Rowe et al 2009 [71] |
| Public sector health facilities | Proportion of RDT positive and negative patients prescribed ACTs | Pre-post cluster randomised trial | 3 districts | Kenya | Skarbinski et al 2009 [72] |

Note

1 attribution of nets refers to the use of the source (colour, type) of net to attribute outputs or outcomes to a defined delivery system.

2 No control was included within the cross sectional surveys. These were conducted alongside a case control study which had an objective of assessing the effectiveness of ITNs rather than the delivery system *per se*.

3 Historical internal control: questions were asked on pre-campaign ownership of ITNs in a post campaign survey and where use is reported on use of a campaign net
